# Allelopathic effect of the methanol extract of the weed species-red sorrel (*Rumex acetosella* L.) on the growth, phytohormone content and antioxidant activity of the cover crop - white clover (*Trifolium repens* L.)

**DOI:** 10.1186/s12870-024-05240-z

**Published:** 2024-06-10

**Authors:** Ho-Jun Gam, Md. Injamum-Ul-Hoque, Yosep Kang, S. M. Ahsan, Md. Mahadi Hasan, Shifa Shaffique, Sang-Mo Kang, In-Jung Lee

**Affiliations:** 1 Department of Applied Biosciences, Kyungpook National University, Daegu, 41566 Korea; 2 Department of Plant Medicals, Andong National University, Andong, Republic of Korea; 3 State Key Laboratory of Herbage Improvement and Grassland Agro-Ecosystems, College of Ecology, Lanzhou University, Lanzhou, Gansu, 730000 China; 4 Institute of Agricultural Science and Technology, Kyungpook National University, Daegu, 41566 Republic of Korea

**Keywords:** Allelopathy, Allelochemical, Antioxidant activity, Phytohormones, Reactive oxygen species

## Abstract

Allelopathy is a biological process in which one organism releases biochemicals that affect the growth and development of other organisms. The current investigation sought to determine the allelopathic effect *of Rumex acetosella* on white clover* (Trifolium repens)* growth and development by using its shoot extract (lower IC_50_ value) as a foliar treatment. Here, different concentrations (25, 50, 100, and 200 g/L) of shoot extract from *Rumex acetosella* were used as treatments. With increasing concentrations of shoot extract, the plant growth parameters, chlorophyll and total protein content of *Trifolium repens* decreased. On the other hand, ROS, such as O_2_^.−^ and H_2_O_2,_ and antioxidant enzymes, including SOD, CAT, and POD, increased with increasing shoot extract concentration. A phytohormonal study indicated that increased treatment concentrations increased ABA and SA levels while JA levels were reduced. For the identification of allelochemicals, liquid‒liquid extraction, thin-layer chromatography, and open-column chromatography were conducted using *R. acetosella* shoot extracts, followed by a seed bioassay on the separated layer. A lower IC_50_ value was obtained through GC/MS analysis. gammaSitosterol was identified as the most abundant component. The shoot extract of *Rumex acetosella* has strong allelochemical properties that may significantly impede the growth and development of *Trifolium repens*. This approach could help to understand the competitive abilities of this weed species and in further research provide an alternate weed management strategy.

## Introduction

Exotic plants are a significant threat to biodiversity, as they disrupt ecosystems by outcompeting native species They have traits that give them a competitive edge, such as fast growth, high reproductive rates, and tolerance to various environmental conditions. Exotic plants also release chemicals that inhibit the growth of native plants, further enhancing their dominance [1].

Allelopathy involves the incorporation of allelochemicals produced by one organism that inhibit or promote the growth of another. Allelopathic plants in intercropping settings produce allelochemicals through root exudates, Volatile organic compound emissions from above-ground parts, and leaching or decomposition of plant detritus [2]. Allelochemicals are secondary metabolites generated as byproducts of plant physiological activities [3]. When they are released, they can impede germination, growth, and development, creating imbalances in the levels of certain phytohormones and reducing root and shoot length, resulting in the degradation of photosynthetic pigments, cell membrane damage, denaturation and the inhibition of protein synthesis [4–8].

Allelochemicals disturb the reactive oxygen species (ROS)–antioxidant equilibrium and modify the physiological state of plants [9]. Plants generate ROS, comprising superoxide anion radical (O_2_^.−^), hydrogen peroxide (H_2_O_2_), hydroxyl radical (OH^−^), and singlet oxygen (^1^O_2_), which are crucial signalling molecules in response to environmental stressors [10, 11]. However, it may undergo scavenging as a result of enzymatic activity, including that of catalase (CAT), superoxide dismutase (SOD) and class III peroxidases (PODs) [12].

*Rumex acetosella* is a exotic weed found in grasslands, pastures, waste areas, and along roadsides [13–15]. It is considered one of the world’s worst weeds, infesting 45 different crops in 70 countries [16]. It is susceptible to shading by other plants [17], but heavy grazing can reduce shading and allow it to compete with native forage grasses [14]. Additionally, it recovers quickly from clipping treatments, which may explain its abundance in grasslands and pastures [18]. It is the predominant exotic species in domestic agriculture in several provinces in South Korea, including Gyeonggi, Gangwon, Chungcheongbuk, Chungcheongnam, Jeollabuk, Jeollanam, and Jeju [19]. The presence of *R. acetosella* has been shown to have a negative impact on both the production and quality of grass in these areas. According to Cooper et al. (1985) [20], the consumption of substantial quantities of *R. acetosella* by cattle leads to the production of oxalic acid-calcium chelate or induces calcium deficiency, resulting in fatality.

White clover (*Trifolium repens*) is a leguminous plant that grows in temperate regions and is known for its high-protein forage [21] and its ability to fix nitrogen at a high rate [22]. Historically, white clover has been used in crop rotations to maintain yields, but its use declined with the introduction of mineral fertilizers in the twentieth century. It is still used in organic systems and in grass/clover leys to improve forage and grassland quality [23, 24]. Recent scholarly investigations have revealed that white clover can serve as living mulch (cover crops) [25], for phytoremediation [26], or as a source of biologically active compounds for protection [27–29]. Frankton and Mulligan [30] reported that a large soil seed bank of *R. acetosella* can also result in crop failure for clover. Few studies have investigated the allelopathic interaction between *Rumex acetosella* and *Trifolium repens* [31, 17, 32–34]. This research aimed to explore the allelopathic effects of the plant *R. acetosella* on *T. repens*. The objective of this study was to determine the mechanisms of allelochemical-plant interactions and use this understanding to develop an environmentally friendly weed control strategy.

## Materials & methods

### Collection of plant material

The *R. acetosella* plant material used in the experiment was collected in May 2022 from Yugyum-ri, Gangdong-myeon, Gyeongju-si, and Gyeongsangbuk-do (35°59′15''N, 129°16′34''E). The specimen was later identified by Professor Dr. In-Jung Lee and deposited into the National Institute of Agricultural Sciences with the deposition number HCCN-2020–4 (for plant) and WS000589 (for seeds). First, the collected plant material was subjected to freeze-drying using a freeze dryer (PVTFD20R, Ilshin Lab, Seoul, Korea). Subsequently, the freeze-dried sample was divided into shoot and root parts. The segments were then finely pulverized using a homogenizer (29000A0, IKA, Staufen, Germany). *T. repens* was purchased from Dongguk Seedling Industry Inc. (Dongguk Seedling White Clover Seed Landscape, Dongguk Seedling Industry Inc., Seoul, Korea).

### Preparation of the methanol extract

To prepare the samples, 500 g dry weight of crushed *R. acetosella* shoot and root were separately placed in a 2 L Erlenmeyer flask (FK10202000, Dongsung Science Inc., Gwangju, Korea). Within each flask, 1 L of methanol (MeOH) was added, and the mixture was stirred using a magnetic stirrer (MSH-20D, DAIHAN Scientific Inc., Wonju-si, Gangwon-do, Korea) for a period of 24 h. After that, filter paper (Advantec No. 2, Toyo Roshi Kaisha Inc., Tokyo, Japan) was positioned on a Büchner funnel to obtain the methanol extract, and this process was repeated three times to ensure thorough extraction. The methanol extracts were placed in a recovery flask, and concentrated extracts were obtained through the use of a rotary evaporator (Eyela Rotary Vacuum Evaporator NN series, Eyela, Tokyo, Japan). Then, 100 mL of distilled water was added. The resulting sample was stored in a deep freezer (IU2386D, Thermo Fisher Scientific, Marietta, OH, United States) by aliquoting 20 mL into a conical tube (50 mL, SPL Life Science, Pocheon, Korea). Then, the sample was dried using a freeze dryer. Finally, the 39 g of dried samples were stored in a refrigerator at 4 °C [35].

### In vitro seed bioassay

An experiment was conducted to investigate the effect of methanol extracts derived from both the shoot and root parts of *R. acetosella* on the germination of *T. repens* seeds through a seed bioassay. To ensure the sterilization of *T. repens* seeds, a solution of sodium hypochlorite (3%) in dH_2_O was used. Subsequently, 20 sterilized seeds were evenly spread on Petri dishes (60 mm × 15 mm, SPL Life Science, Pocheon, Korea) covered with filter paper. Two stock water solutions, each with a concentration of 20 g/L, were prepared using the shoot and root extracts *of R. acetosella*. Serial dilutions were performed to create a range of concentrations for the experiment, including 20, 10, 5, 2.5, 1.25, and 0.625 g/L. After the addition of 1 mL of methanol extract to a Petri dish with seeds, the plants were grown for a period of seven days in a controlled plant growth chamber (JSPC-420C, JSR Corporation, Gonju, Korea) at a temperature of 20 °C, humidity of 60%, and light intensity of 6850 lx. The growth parameters were carefully investigated during the growth period. A dose‒response curve was obtained based on the measured fresh weight, allowing for the calculation of the IC_50_ value, which represents the concentration of the extract at which plant growth is inhibited by 50%. This experiment was repeated three times to ensure the reliability of the results.

### Foliage treatment and chlorophyll content measurement

*T. repens* seeds were subjected to sterilization through a sodium hypochlorite (3%) solution, and subsequently, 20 seeds were planted in separate 100 mm × 90 mm pots containing cocopeat (68%), perlite (11%), zeolite (8%), as well as micronutrients available as NH_4_^+^∼0.09 mg/g; P_2_O_5_∼0.35 mg/g; NO_3_^−^∼0.205 mg/ g; and K_2_O ∼0.1 mg/ g. After preparing the stock solution of the *R. acetosella* shoot extract at a concentration of 200,000 mg/L, serial dilution was carried out, and the adjuvant Tween20 (0.01%) (Duksan Genetal Science Inc., Seoul, Korea) was added. The concentrations used in the experiment were 200 g/L, 100 m/L, 50 g/L, 25 g/L. Following a 14-day growth period (Trifoliate stage) from the time of sowing, foliar treatments were administered three times, each at weekly intervals with a 5 mL volume. Three days after the final foliar treatment, various plant growth parameters, including shoot length, root length, fresh weight, and dry weight, were assessed. The chlorophyll content was measured with a portable chlorophyll content metre (CCM-300, ADC Bioscientific Inc., Herts, UK).

### Measurement of total protein content

To determine the total protein content, 0.1 g of shoot fresh sample was ground with liquid nitrogen and mixed with 1 mL of 100 mM sodium phosphate buffer (pH 7.0) in an E-tube. The mixture was then centrifuged at 12,000 × g for 30 min, and the protein content of the supernatant was analyzed at 595 nm using a spectrometer, following established methods [36].

### Determination of ROS activity

The O_2_^−^ content was measured using the method reported by Navari-Izzo et al. in 1999 [37]. 0.1 g of freshly ground shoot sample was used, then 10 mM NaN_3_ solution, 0.05% NBT, and 10 mM potassium phosphate buffer (pH 7.8) were mixed, and they were agitated for some time. After chilling and centrifugation, the obtained solution was heated to about 85 °C for 15 min. The level of O_2_^−^ activity in the aqueous fraction was determined using a spectrophotometer at wavelength of 580 nm. The effect of H_2_O_2_ in *T. repens* leaves treated with *R. acetosella* extract was examined with a commercial assay kit. The sample then was frozen in liquid nitrogen. After that, 0.1 g of fresh phosphate was added to the ice-cold phosphate buffer solution (pH 7.8) that included 1 mM EDTA. The supernatant obtained after centrifugation was analysed using an OxiTec™ Hydrogen Peroxide/Peroxidase Assay Kit. The H_2_O_2_ Assay Kit uses oxiprobe and peroxidase (POD) as enzyme pairs to enable the quantification of hydrogen peroxide (H_2_O_2_) activity.

### Determination of antioxidant activity

The assessment of antioxidant enzymes in *T. repens* leaves after treatment with *R. acetosella* extract involved using a commercial test kit. A 0.1 g fresh weight of leaves sample was ground with liquid nitrogen in an E-tube and combined with 1 mL of 50 mM phosphate buffer pH 7.8 and 1 mM EDTA. The mixture was vortexed, ice-incubated, and then centrifuged. The upper cell culture supernatant layer was used for OxiTec™ SOD, Catalase, and Hydrogen Peroxide/Peroxidase Assay Kits from Biomax Co., Ltd., following specified techniques [38]. The SOD Assay Kit analyzes SOD activity via xanthine oxidase and WST, with a spectrophotometer reading at 450 nm indicating enzyme activity. The CAT Assay Kit uses oxiprobe, horseradish peroxidase, and catalase to measure catalase activity by reacting H_2_O_2_ with catalase to produce water and oxygen. Unconverted H_2_O_2_ reacts with oxiprobe and peroxidase to form resorufin, which can be measured at 560 nm using a spectrophotometer [39]. There was an inverse relationship between the CAT activity and the observed absorbance. The POD Assay Kit uses an oxiprobe and hydrogen peroxide (H_2_O_2_) to quantify peroxidase (POD) activity [40].

### Quantification of abscisic acid (ABA)

Endogenous ABA in plants was quantified using the technique described by [41]. A 0.1 g dry weight of leaves of *T. repens* sample was mixed with 10 mL of ABA extraction solvent (95:5 isopropanol and acetic acid). After 30 min, the mixture was filtered and concentrated under reduced pressure with the addition of 100 ng of the [( ±)-3,5,5,7,7,7-d6] ABA standard. The residue was dissolved in 1 N NaOH, and the pH was adjusted to 12–13. Chlorophyll was removed with CH_2_Cl_2_, and the pH of the supernatant was adjusted to 2.5–3.5. Ethyl acetate (EtoAC) was added, and the solution was concentrated under reduced pressure. After dissolution in pH 8.0 phosphate buffer, the solution was mixed with 1 g of PVPP and filtered, and the pH was adjusted to 2.5–3.5. The supernatant was collected, concentrated, and dried with N_2_ gas. The samples were methylated with diazomethane, dissolved in 50 µL of CH_2_Cl_2_, and analysed by GC/MS after injecting 1 µL of each sample.

### Quantification of jasmonic acid (JA)

Endogenous jasmonic acid (JA) in plants was quantified using the method outlined by [42]. A solution of extracted JA (acetone and 50 mM citric acid, 70:30, v/v) was added to 0.3 g of dry weight of leaves of *T. repens* and agitated for 30 min. The [9,10-2H_2_]-9,10-dihydro-JA standard was then added, and the mixture was filtered using a Buchner apparatus and filter paper. The resulting solution was concentrated under reduced pressure and dissolved in 100 mM phosphate buffer (pH 7.5), and the pH was adjusted to 2.5. The sample was treated with diethylaminoethyl cellulose (DEAE cellulose) and shaken for one hour before being filtered too. The bottom layer was separated by chloroform and transferred into an open column containing of anhydrous NaSO_4_ to get rid of the remaining water. The liquid was concentrated by reducing the pressure. The final residue was dissolved with ethyl ether and then transferred to an amino cartridge (Grace Pure™ SPE Amino, Grace Dev, IL, USA). Contaminants were removed using a solution of chloroform and isopropanol (2:1) and a mixture of ethyl ether and acetic acid (49:1). In the end, solution was decanted, evaporated and concentrated to the necessary concentration under reduced pressure. The residual part was dissolved in ethyl ether, put into a vial, and dried up with N_2_ gas. The samples were methylated by the use of diazomethane, dissolved in anhydrous CH_2_Cl_2_ and analyzed by the combination of gas chromatography and mass spectrometry (GC/MS).

### Quantification of salicylic acid (SA)

The endogenous salicylic acid (SA) in the plants was quantified by slight changes of the method described in [42]. A solution consisting of 90% MeOH was used to the sample that contained 0.1 g of dry weight of leaves of *T. repens*. After sonication of the mixture it was transferred to an E-tube. Afterwards, centrifugation was carried out at 12,000 rpm for 15 min at 4 °C. After the supernatant collection, 100% methanol (MeOH) solution was added and the process was continued. The concentration of the supernatant was performed on a speedvac (model SPD2030, Thermo Fisher Scientific, Waltham, MA, USA). Finally, 5% trichloroacetic acid was placed in the obtained residue and the resulting mixture was transferred to E-tube with subsequent centrifugation. Following the separation of the supernatant, an extraction solution consisting of ethyl acetate, cyclopentane, and isopropanol at a volumetric ratio of 49.5:49.5:1 (v/v/v) ratio was applied. Subsequently, the supernatant was subjected to separation. The supernatant, which included the SA component, was thoroughly dried using nitrogen gas (N_2_). The residue was dissolved in 1 mL of injection solution consisting of 100% methanol (MeOH), and a volume of 20 µL was thereafter injected into the high-performance liquid chromatography (HPLC) system for the purpose of quantitative analysis.

### Identification of allelochemicals from the shoot extract of *Rumex* acetosella

#### Liquid‒liquid extraction (LLE)

LLE was performed with the methanol extract of the shoot of *R. acetosella*, which was freeze-dried. After 40 g of the sample was dissolved in 400 mL of distilled water, it was placed in a separate funnel. In the case of solvents, n-hexane, dichloromethane (CH_2_Cl_2_), chloroform (CHCl_3_), and ethyl acetate (EtOAc) were selected because of their miscibility and polarity. The same amount of solvent as the sample dissolved in distilled water was added to a separate funnel, and LLE was performed three times. After filtering with anhydrous sodium sulfate (NaSO_4_) to completely remove water from the solvent, the mixture was concentrated in a recovery flask using a vacuum concentrator. After freeze-drying, each concentrated layer was subjected to the seed bioassay described above. As a result of the seed bioassay, the n-hexane layer with the lowest IC_50_ value was used in the next process.

#### Thin Layer Chromatography (TLC)

Thin layer chromatography (TLC) was performed to determine the ratio of the mobile phase developing solvent used for the first and second column chromatographies. TLC was performed on 60G silica gel F_254_ 25 glass plates (2020 cm) [43].

#### Column chromatography

First, column chromatography was performed to separate substances with allelopathy potential from the n-hexane layer, which had the lowest IC_50_ value among the solvent fractions. A mixed solution of n-hexane–ethyl acetate (29:11, v/v) was used as the mobile phase, and 5 mL of each mixture was subjected to elution fractionation by chromatography (silica gel 60, 0.040–0.063 mm, Merck). After fractionation, TLC was performed as described above, and the samples were separated into the following fractions: n-hexane layer fraction A (HA), n-hexane layer fraction B (HB), n-hexane layer fraction C (HC), n-hexane layer fraction D (HD), and n-hexane layer fraction E (HE). After concentrating each fraction, a seed bioassay was performed, and secondary column chromatography was performed on the HD fraction with the lowest IC_50_ value. Before proceeding with the second chromatography, the mobile phase was composed of an n-hexane-dichloromethane-ethyl acetate mixed solution (29:8:11, v/v/v) through TLC, and 5 mL of each was subjected to elution fractionation by chromatography. After fractionation, TLC was performed as described above, and the samples were separated into the n-hexane layer fraction DA (HDA), n-hexane layer fraction DB (HDB), n-hexane layer fraction DC (HDC), n-hexane layer fraction DD (HDD), and n-hexane layer fraction DE (HDE) from the n-hexane layer fraction D (HD). After concentrating each fraction, a seed bioassay was performed for each fraction, and among them, instrumental analysis was performed for the HDC fraction with the lowest IC_50_ value [44].

#### Instrumental analysis

The HDC fraction was dissolved in n-hexane at a concentration of 25 mg/0.5 ml and then analysed by gas chromatography‒mass spectrometry (GC/MS) with a scan system (7890B Network GC System and 5977B Network Mass Selective Detector; Agilent Technologies, Palo Alto, CA, USA).

### Statistical analysis

The experiments were repeated three times. Statistical analysis (One-way ANOVA) was performed using SAS On Demand for Academics (Version 3.1.0, SAS Institute Inc., CARY, North Carolina, USA), with significance tested at *p* < 0.05 using Duncan’s multiple range test (DMRT). IC50 values were calculated in GraphPad Prism 5 (version 5, GraphPad Software, San Diego, CA, USA) using log[inhibitor] vs. normalized response—variable slope in dose‒response – inhibition.

## Results

### In vitro seed bioassay

**Fig. 1:**
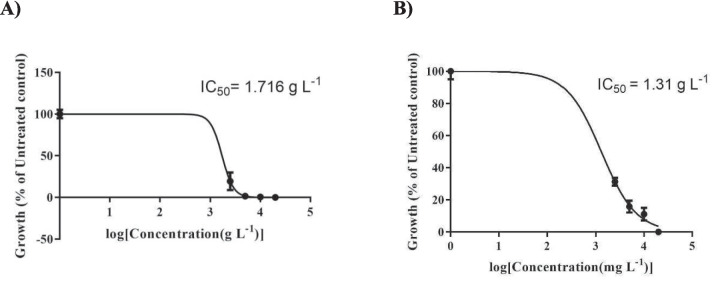
IC_50_ values of the **A** Root and **B** Shoot Extracts of *R. acetosella*

To conduct the in vitro seed bioassay, we measured the half-maximal inhibitory concentration (IC_50_) (in g/L) for *T. repens* germination and growth using extracts from both the shoot and root parts of *R. acetosella* (Fig. 1). The IC_50_ values obtained were 1.72 g/L for the root extract and 1.31 g/L for the shoot extract. Therefore, the subsequent foliage treatment experiment was carried out exclusively with the shoot extract.

### Measurement of growth parameters

**Fig. 2:**
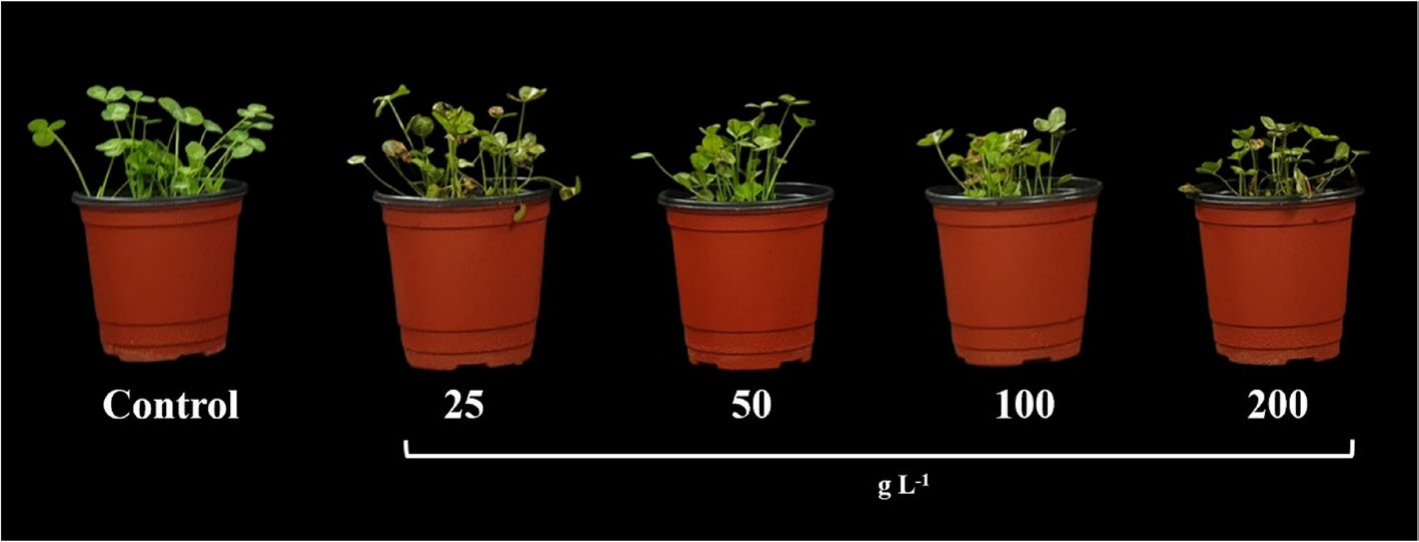
The impact of varying concentrations of *R. acetosella* shoot extract on the growth parameters of *T. repens*

The results revealed a notable variation in the impact of *R. acetosella* extract at different concentrations on the morphological attributes of *T. repens* (Fig. 2). Specifically, for shoot length, no significant differences were observed among the groups treated with the concentrations of 25, and 50 g/L when compared to the control group. In contrast, significant differences were observed in shoot length at concentrations of 100 and 200 g/L. In terms of root length, the application of *R. acetosella* extract at different concentrations did not significantly affect root length. Furthermore, no significant differences in fresh weight were observed at concentrations of 25 g/L compared to the control group. Nonetheless, significant differences were detected at concentrations of 50, 100, and 200 g/L (Table 1).

**Table 1:** Effect of different concentrations of *R. acetosella* shoot extract on the growth parameters of *T. repens*. The values indicate the means ± SDs for 3 replications

Morphological Parameters	Control	25 (gL^−1^)	50 (gL^−1^)	100 (gL^−1^)	200 (gL^−1^)
Shoot length (cm)	7.3 ± 0.35^a^	7.2 ± 0.22^ab^	6.4 ± 0.42^ab^	5.9 ± 0.36^bc^	4.9 ± 0.66^c^
Root length (cm)	12.5 ± 0.47^a^	12.1 ± 0.58^a^	11.3 ± 0.18^a^	11.2 ± 0.24^a^	10.7 ± 0.28^a^
Fresh weight (g)	0.55 ± 0.06^a^	0.46 ± 0.12^ab^	0.33 ± 0.03^bc^	0.32 ± 0.12^bc^	0.21 ± 0.03^c^

Different letters denote significant differences, while similar letters denote nonsignificant differences among the treatments. Columns labelled with different letters indicate significant differences at the *p* ≤ 0.05 level

### Determination of chlorophyll content

**Fig. 3:**
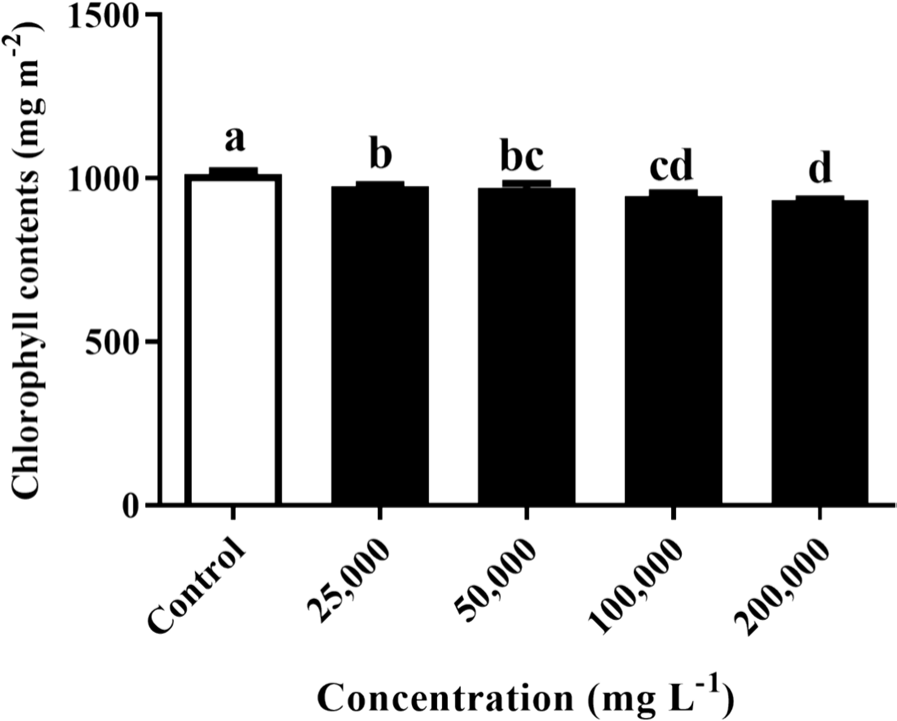
Effect of different concentrations of *R. acetosella* shoot extract on the chlorophyll content of *T. repens*. The values indicate the means ± SDs for 3 replications. Bars labelled with different letters indicate significant differences at the *p* ≤ 0.05 level

The impact of varying concentrations of *R. acetosella* extract on the chlorophyll content is visually represented in Fig. 3. Notably, as the concentration of the shoot extract increased, there was a consistent reduction in chlorophyll content across all treatments. Significantly, compared with those in the control group, the chlorophyll content in all treatment groups substantially decreased. With each increase in extract concentration, the chlorophyll content decreased by 3.9%, 4.3%, 6.8%, and 7.9%, respectively, in comparison to that in the control.

### Visualization of ROS (H_2_O_2_)

**Fig. 4:**
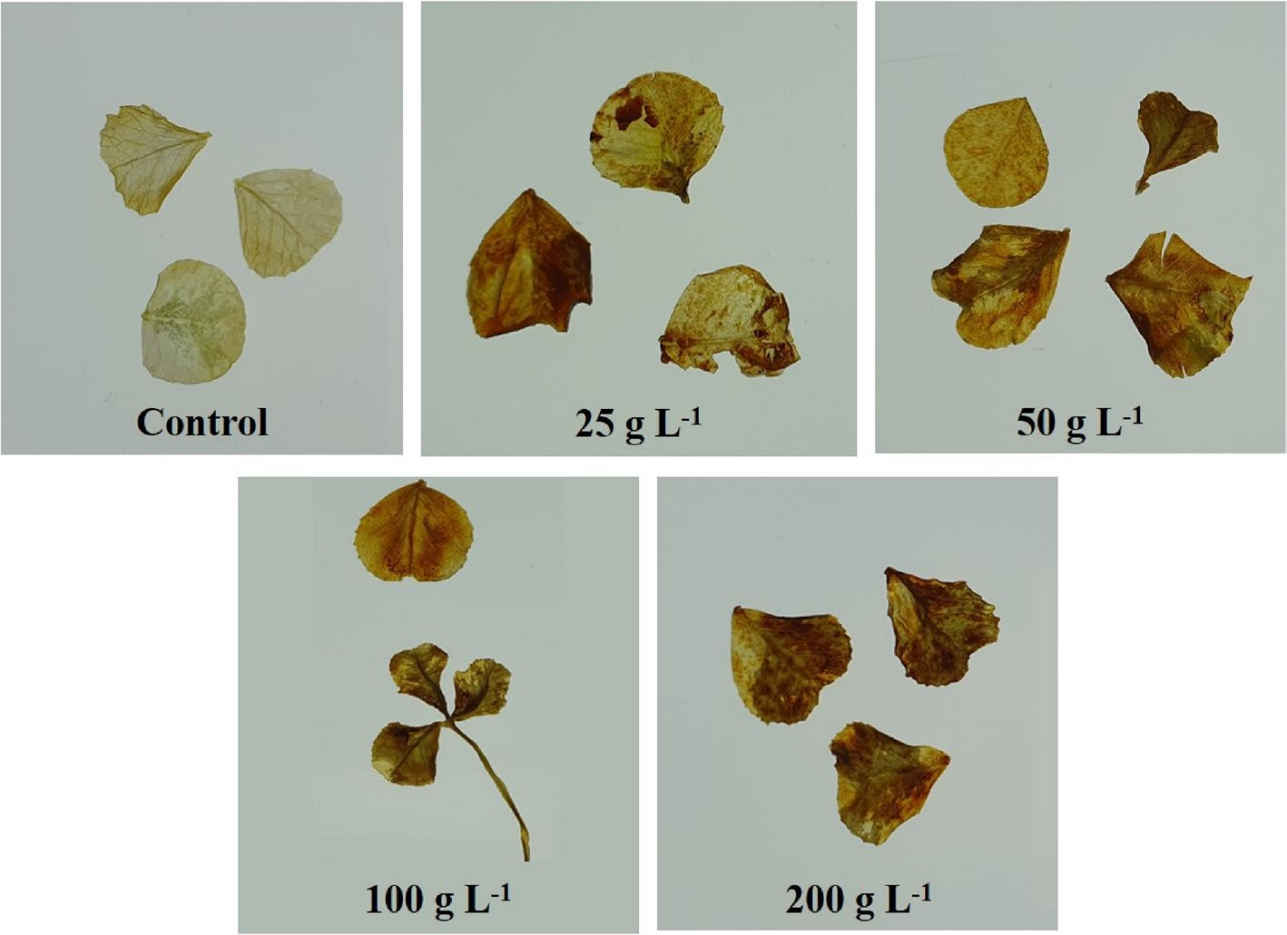
Effect of *R. acetosella* extract treatment on the H_2_O_2_ content of *T. repens*

H_2_O_2_ was detected by staining the mature leaves of *T. repens* with 3,3′-diaminobenzidine (DAB). This staining process relies on the oxidation of DAB by hydrogen peroxide, especially in the presence of certain heme-containing proteins, such as peroxidases, resulting in the formation of a distinct dark brown precipitate. In contrast to the control group, the foliar treatment groups exhibited brown staining, indicating a substantial presence of H_2_O_2_ in the mature leaves of *T. repens* (Fig. 4).

### Determination of hydrogen peroxide (H_2_O_2_) and superoxide anion (O_2_^−^) activity

**Fig. 5:**
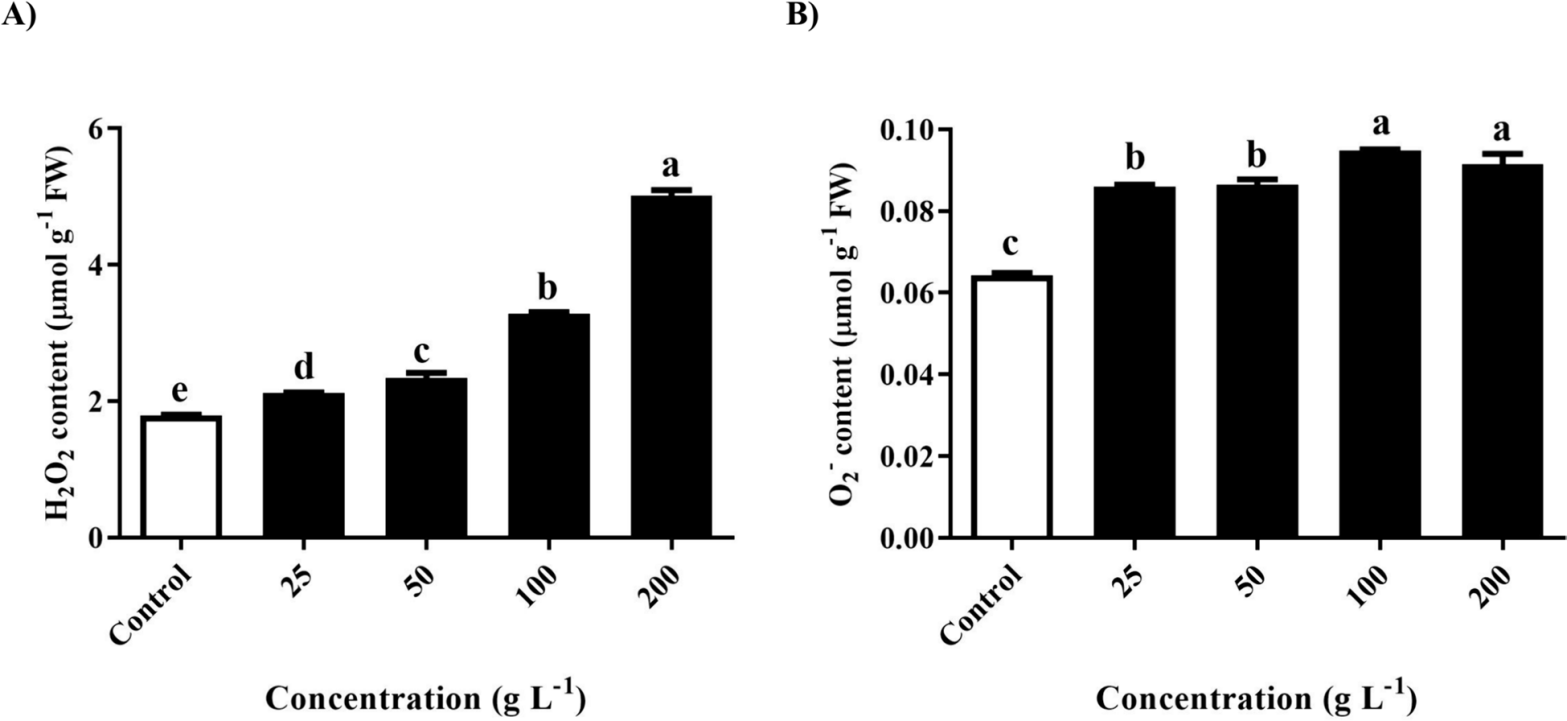
Measurement of the of **A** hydrogen peroxide (H_2_O_2_) and **B** superoxide anion (O_2_^−^) in *T. repens* leaves after treatment of different concentrations of shoot extracts of *R. acetosella*. The values indicate the means ± SDs for 3 replications. Bars labelled with different letters indicate significant differences at the *p* ≤ 0.05 level

These results revealed the intricate relationships between foliar treatment with different concentrations of *R. acetosella* extract and foliar H_2_O_2_ and O_2_^−^content. As shown in Fig. 5 (A), the H_2_O_2_ content significantly increased in all treatment groups compared to that in the control group. With the increase in extract concentration to 25, 50, 100, and 200 g/L, the H_2_O_2_ content increased by 18.4%, 31.2%, 84.1% and 180.9%, respectively. These results clearly indicate that as the concentration of extract increases, so does the activity of hydrogen peroxide (H_2_O_2_) within the foliar tissue.

Conversely, a comparable trend was observed in the superoxide anion (O_2_^−^) activity compared to that of the control (Fig. 5B). After the application of the foliar extract of *R. acetosella*, there was a notable increase in the activity of the superoxide anion (O_2_^−^). This increase was measured at 33.6%, 34.4%, 47.5%, and 42.4%, corresponding to the incremental concentration of the extract, respectively. These findings underscore a consistent increase in superoxide anion (O_2_^−^) activity with increasing concentrations of the *R. acetosella* extract.

### Measurement of total protein content

**Fig. 6:**
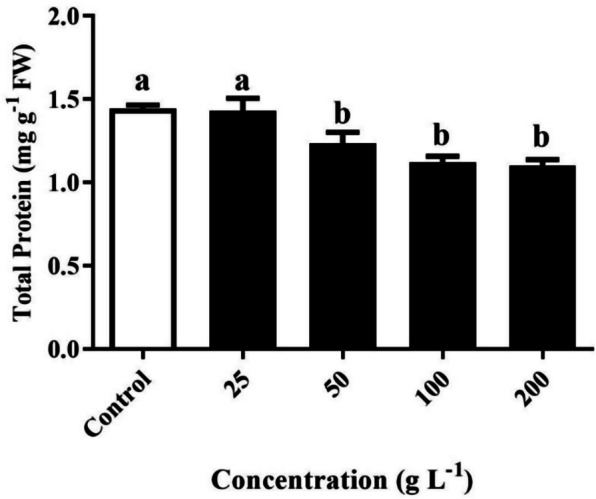
Measurement of the total protein content of *T. repens* treated with different concentrations of *R. acetosella* extract. The values indicate the means ± SDs for 3 replications. Bars labelled with different letters indicate significant differences at the *p* ≤ 0.05 level

The application of *R. acetosella* extract through foliage treatment at various concentrations had a noteworthy impact on the total protein content (Fig. 6). Except at 25 g/L, a significant decrease in total protein content was evident in all treatment groups compared to that in the control group. The total protein content decreased by 0.9%, 14.5%, 22.3%, and 23.6% in the respective treatment groups compared to that in the control group. These data underscore the considerable reduction in total protein content with increasing *R. acetosella* extract concentration.

### Measurement of antioxidant activities

**Fig. 7:**
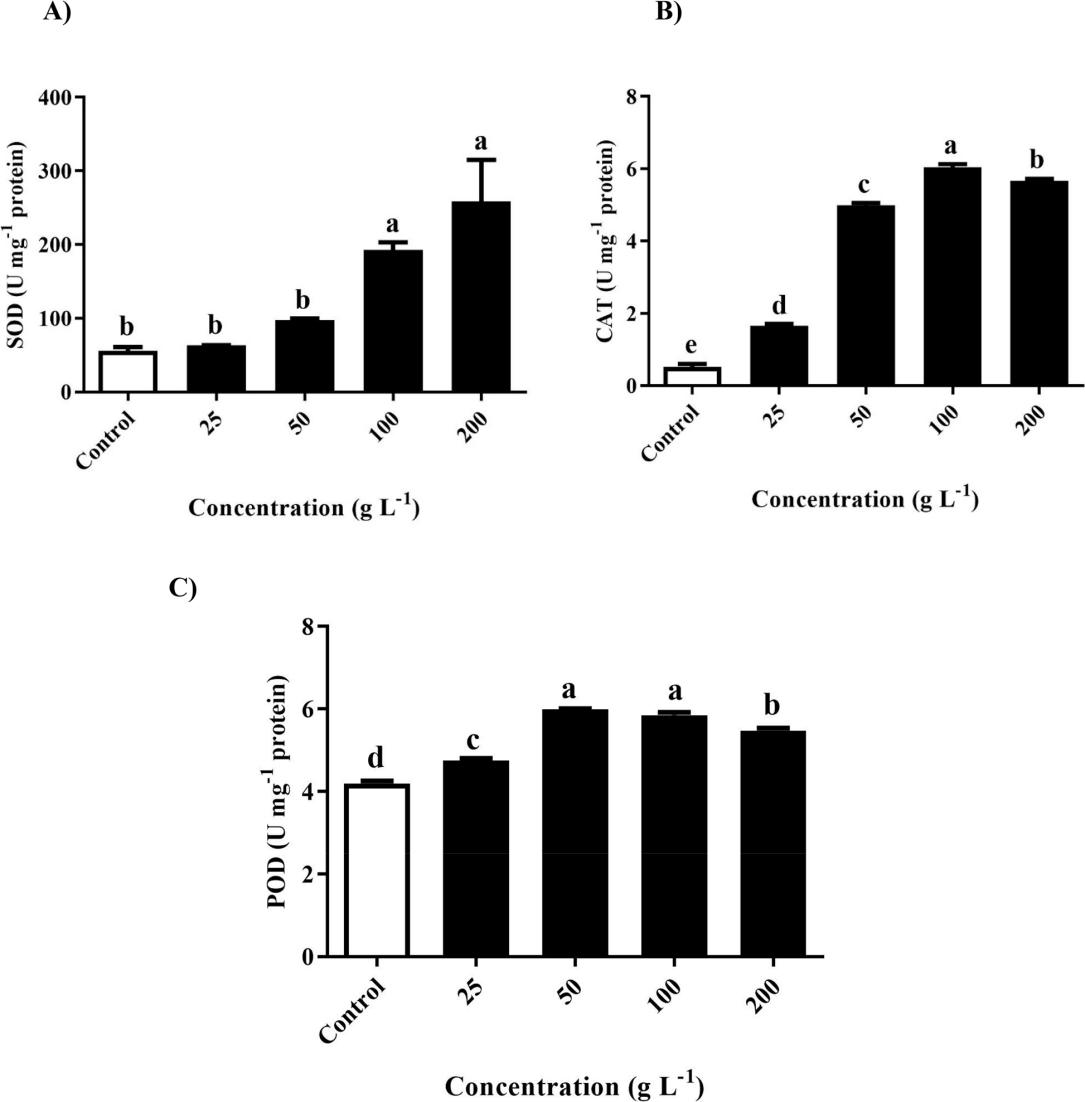
Effect of different concentrations of *R. acetosella* shoot extract on the activity of **A** SOD, **B** CAT and **C** POD in *T. repens* leaves*.* The values indicate the means ± SDs for 3 replications. Bars labelled with different letters indicate significant differences at the *p* ≤ 0.05 level

Plants rely on their antioxidant systems to protect themselves against the cytotoxic effects of environmental stressors. Critical enzymes, including superoxide dismutase (SOD), catalase (CAT), and peroxidase (POD), play pivotal roles in enhancing plant resistance to free radical damage. The figure shows insights into the measurement of SOD activity within *T. repens* following exposure to foliar extracts of *R. acetosella* at various concentrations (Fig. 7A). Notably, there were no significant differences observed between the control group and the 25 g/L and 50 g/L treatment groups. However, a notable trend emerged as the concentrations increased, with the 100 and 200 g/L treatments displaying significantly greater SOD activity than the control. This observation revealed the potential of *R. acetosella* extract to enhance SOD activity, particularly at relatively high concentrations.

The activity of catalase significantly increased in *T. repens* leaves after treatment with *R. acetosella* foliar extract across all concentrations, as depicted in Fig. 7B. This highlights the ability of the extracts to enhance catalase activity, contributing to improved antioxidant protection. A similar trend was observed for POD activity in *T. repens* after treatment with various concentrations of *R. acetosella* extract (Fig. 7C). Compared with those in the control group, all treatment groups exhibited significantly greater POD activity at various concentrations, with increases of 13.4%, 42.9%, 39.4% and 30.5%, respectively. This indicates that the extracts can effectively boost POD activity, bolstering plant antioxidant defenses.

### Determination of phytohormones

**Fig. 8:**
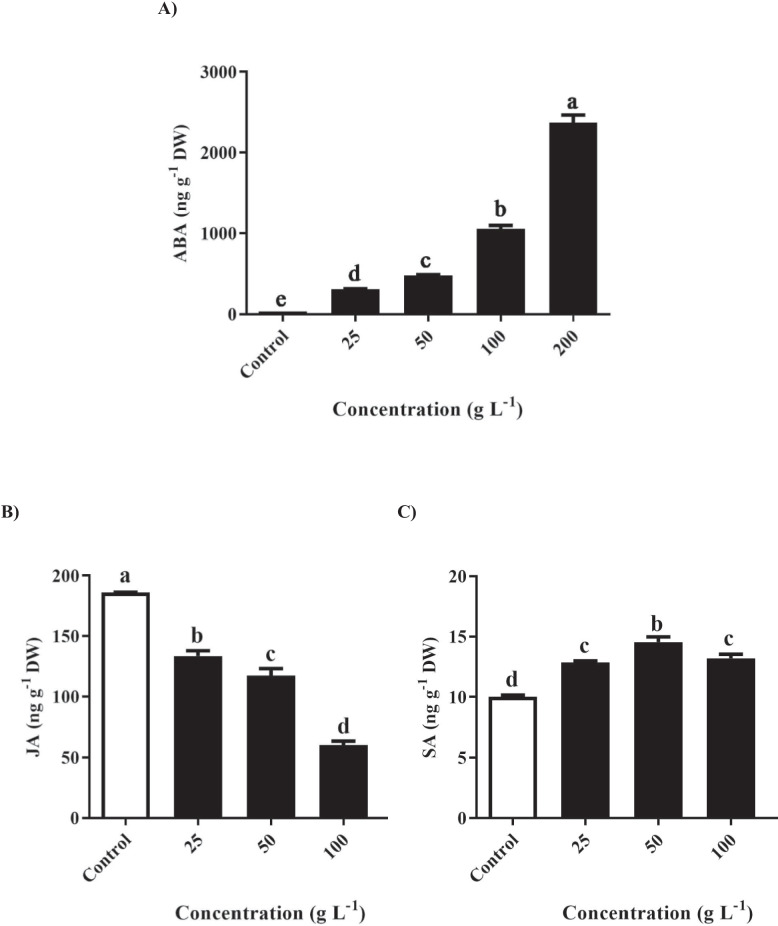
Quantification of phytohormones such as **A** abscisic acid (ABA), **B** jasmonic acid (JA) and **C** salicylic acid (SA) using different concentrations of shoot extracts of *R. acetosella.* The values indicate the means ± SDs for 3 replications. Bars labelled with different letters indicate significant differences at the *p* ≤ 0.05 level

Different concentrations of *R. acetosella* extract had varying effects on endogenous ABA, JA, and SA in *T. repens* leaves. The ABA content significantly increased at 25, 50, 100, and 200 g/L in response to foliage treatment with *R. acetosella* extract (Fig. 8A). In contrast, the JA content significantly decreased in all treatment groups compared to that in the control group, with no significant differences between the 100 g/L and 200 g/L treatment groups (Fig. 8B). These findings highlight the influence of the extracts on ABA and JA levels in *T. repens* leaves.

On the other hand, when *T. repens* was subjected to foliage treatment with *R. acetosella* extract at varying concentrations, the SA content significantly increased in all treatment groups compared to that in the control group. This finding underscores the ability of the extracts to increase the SA content within *T. repens*, thereby contributing to a notable alteration in its physiological process (Fig. 8C).

### Identification of allelochemicals in the shoot extracts of *R. acetosella*

#### Identification through LLE (liquid‒liquid extraction)

LLE was performed as described above, and after the concentration of each fraction, the seed bioassay was performed. The IC_50_ values were 1.187 g L^−1^ for the n-hexane fraction, 1.798 g L^−1^ for the dichloromethane fraction, and 1.503 g L^−1^ for the EtOAc fraction. Therefore, the first column chromatography was performed with the n-hexane fraction, which had the lowest IC_50_ value, except for the chloroform fraction, which had an ambiguous value.

Seed bioassays were performed on the HA, HB, HC, HD, and HE fractions separated by primary column chromatography. Among them, secondary column chromatography was performed on the HD fraction, which had the lowest IC_50_ value of 0.4189 g/L.

**Fig. 9:**
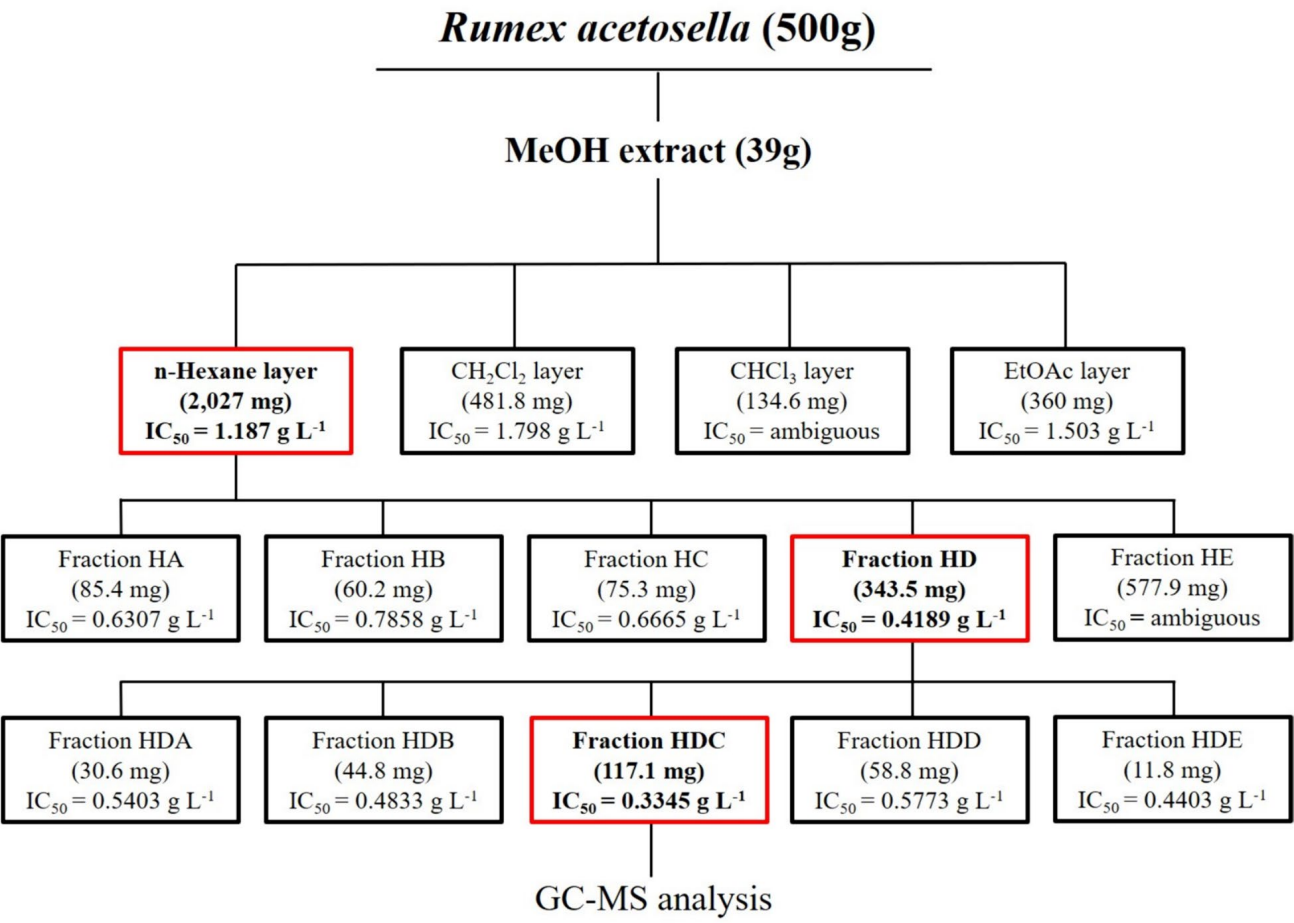
Isolation and identification of allelochemicals from shoot extracts of *R. acetosella*

Seed bioassays were performed on the HDA, HDB, HDC, HDD, and HDE fractions separated by secondary column chromatography. Among them, GC/MS analysis was performed on the HDC layer, which had the lowest IC_50_ value of 0.3345 mg/L (Fig. 9).

#### Identification through GC/MS analysis

GC/MS analysis of the HDC layer revealed 6 major compounds with varying peaks, retention times (RTs) and peak areas (in terms of composition percentage), accounting for 91.3% of the overall composition. Among the compounds, gamma. -Sitosterol had the highest peak area of 60.23%, followed by 9,12-octadecadienoic acid (Z, Z)- (18.07%), campesterol (7.50%), and (Z)6(Z)9-pentadecadien-1-ol. (2.23%),.alpha. Amyrin accounted for 1.71%, and stigmastanol accounted for 1.56% (Table 2).

## Discussion

The present study was designed to determine the allelopathic effects of extracts from the shoots and roots of *R. acetosella* on *T. repens*. Allelopathy may be affected by allelochemicals produced by plants under environmental stress [45]. Due to the treatment of *R. acetosella* extracts in which some herbicidal actives, such as catechol and chrysophanic acid [46, 47], have been identified, it was expected that germination and growth would be inhibited. The present study revealed that the IC_50_ values of the root and shoot extracts were 1.716 g L^−1^ and 1.305 mg L^−1^, respectively (Fig. 1). Due to the lower IC_50_ value in the present study, foliar treatment was performed using the shoot extract of *R. acetosella* (Fig. 1). The findings showed that *R. acetosella* shoot extracts allelopathically inhibited *T. repens* seedling growth.

In the present study, shoot length significantly decreased with increasing extract concentration (Table 1). In addition, the root length and fresh weight decreased with increasing shoot extract concentration (Table 1). The present findings are fully consistent with a previous study [48]in which reported that *A. nilotica* leaf extract at a relatively high concentration was toxic and significantly decreased plant growth in pea. In a study by [49], isoliquiritigenin, an allelochemical, inhibited the growth of lettuce plants at concentrations of 0.2–1.0 mM by affecting cell division and growth hormones, resulting in shorter roots and shoots [50]. Allelopathic stress can lead to changes in pH, osmotic capacity, cell injury, membrane permeability, mineral uptake, and water absorption in seedlings, ultimately reducing stem growth [51–53].

The present study showed that the chlorophyll content decreased, as a result of increasing concentrations of shoot extract (Fig. 3). Our results are consistent with those of [54], who reported that aqueous leaf extracts of *K. integrifoliola* reduced the chlorophyll content in *L. perenne* leaves, possibly by inhibiting proteins involved in chlorophyll synthesis [55].

Figure 6 shows a significant decrease in total protein content with different treatments in contrast to the control. The current results support the findings of a previous study [56], which showed that the aqueous leachate and organic fractions of *Nicotiana* reduced the total protein content in the weeds *S. sophera*, *C. album*, *S. tora*, and *S. viridis*. This may be due to the presence of allelochemicals that inhibit protein synthesis, degrade nucleic material, or interfere with cell division [57].

The present investigation revealed that H_2_O_2_ activity and superoxide anion (O_2_^−^) increased with increasing concentrations of the extract (Fig. 5A and 5B). Representative ROS include hydroperoxide (H_2_O_2_), superoxide anion (O_2_^−^), and hydroxyl radical (·OH). Singlet oxygen (^1^O_2_). This result is consistent with a previous study in which the O_2_^−^ content increased as the concentration of the extract increased, which is presumed to be due to oxidative stress caused by allelochemicals [58]. In the present study, SOD, CAT and POD increased with increasing extract concentration (Fig. 7A-C). When ROS levels increase, antioxidants quickly neutralize them [59, 60]. This increase in antioxidants indicates that a plant is under stress, such as in an allelopathic interaction [61].

Moreover, ABA plays a pivotal role in regulating H_2_O_2_ under plant stress conditions, and the H_2_O_2_ content increases as the amount of endogenous ABA in plants increases [62, 63]. The results of the ABA and H_2_O_2_ content analysis experiments showed that the ABA content increased significantly with increasing extract treatment concentration, and the H_2_O_2_ content also increased significantly. These results are typical when considering the relationship between ABA and H_2_O_2_ as well as allelochemical stress.

**Table 2:**
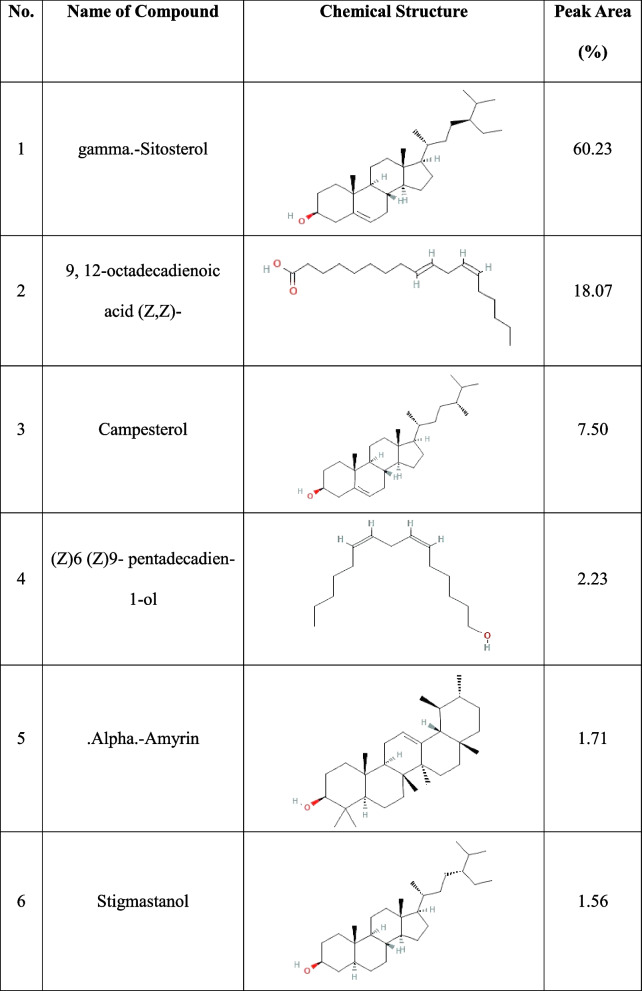
Main components isolated from the HBD fraction of *R. acetosella*

In the present study, endogenous phytohormone analysis revealed that the ABA and SA contents increased with increasing concentrations of *R. acetosella* extract (Fig. 8A, B), while the JA content decreased (Fig. 8C). Similarly, ABA and SA contents increased during foliar treatment with allelochemicals [54, 64]. ABA acts as a signal transmitter in response to abiotic and biotic stress in plants [65, 66], and this stress causes endogenous ABA accumulation in plants. SA also participates in the response to abiotic stress and induces endogenous SA accumulation in response to abiotic stress [67]. It is assumed that ABA and SA accumulation are caused by stress caused by allelochemicals contained in the *R. acetosella* extract. JA is a hormone that acts as a signalling pathway in response to abiotic stresses such as cold, drought, salt, heavy metals, and light [68]. However, unlike those of ABA and SA, the JA content decreased as the treatment concentration increased, presumably because SA and ABA act as antagonists of JA [69, 70].

GC/MS analysis revealed that gamma-sitosterol, which is the most common component in *R. acetosella* extract (Table 2), is an isomer of β-sitosterol. Gamma-Sitosterol is also known to have anti-inflammatory and antidiabetic potential [71, 72].Phytosterols (PSs) are part of the “triterpene” family and are similar to cholesterol in terms of their action and structure. PSs have an additional side chain, unlike cholesterol [73]. In plants, PSs are structural components similar to cholesterol in humans. The two most prevalent PSs, sitosterol and campesterol, contain additional methyl and ethyl groups at position C-24, respectively. Another PS is stigmasterol, which has a double bond at position C-22 and an extra ethyl group at position C-24 [74, 75]. Plant sterols, such as campesterol, sitosterol, and stigmasterol, can be converted into campestanol and sitostanol/stigmastanol when fully saturated. These saturated forms have no double bonds in the steroid nucleus or the alkyl side chain. Plant sterols offer health benefits, including antiobesity, antidiabetic, antimicrobial, anti-inflammatory, and immunomodulatory effects. They may also reduce the risk of cancer by 20% [76]. Therapeutic herbs contain beta-amyrin. Both in vitro and in vivo research have revealed the biological roles of beta-amyrin. The chemistry and pharmacology of amyrins and their analogues have garnered significant attention [77]. Plants produce beta-sitosterol, a white waxy powder, through a biological synthesis pathway. Studies suggest that beta-sitosterol has various pharmacological and therapeutic uses. Gamma-sitosterol is a stereoisomer of beta-sitosterol [78]. It inhibits cell growth, apoptosis, and cell cycle arrest [79]. Docking studies indicate that gamma-sitosterol has a strong binding energy and low inhibition constant, making it a promising candidate for development as a bioactive agent [71, 80–82].

Plants produce a wide variety of fatty acids, which are long linear hydrocarbon chains that are ‘saturated or unsaturated’ with an even number of carbon atoms [83]. 9,12-Octadecadienoic acid (ZZ) is a key fatty acid that plays a crucial role in prostaglandin biosynthesis and has various biological functions, including anti-inflammatory, antihistaminic, antiarthritic, and hepatoprotective effects [84]. Fatty acid alcohols are effective at killing viruses, bacteria, and fungi. Studies have been conducted for more than 50 years to determine whether lipids are involved in the body’s natural defense against pathogens [85]. (Z)6, (Z)9-Pentadecadien-1-ol is an alcohol derived from fatty acids and has antibiotic properties [86]. An appropriate concentration of allelochemicals is crucial for their toxic effects. A concentration that is too high can affect multiple target sites [87]. From the GC‒MS analysis, gamma. -Sitosterol had the highest peak area of 60.23% (Table 2).

## Conclusions

The study’s findings highlight the allelopathic effect of *Rumex acetosella* on the growth of white clover, with gamma-sitosterol identified as a significant inhibitory compound among others in the shoot extracts. This outcome suggests *R. acetosella’s* potential role in allelopathic interaction with white clover. However, given that gamma-sitosterol is a common sterol found in various plant species, and considering that we did not directly test the isolated compound, its specific contribution to the observed allelopathic effects remains to be conclusively determined. Additionally, while we highlighted the fraction with the lowest IC50 value as potentially the most effective, the close range of IC50 values across different fractions suggests a possible combined effect of multiple compounds. In light of these considerations, further investigation involving the isolation and direct testing of gamma-sitosterol and other active compounds within these extracts would be required to accurately quantify their individual allelopathic impacts.

Allelopathic effects of invasive plant allelochemicals, particularly terpenes, have consistently inhibited the growth and development of clover. These compounds may also play a role in detecting neighboring plants and preparing for competition, although the specific mechanisms remain unclear. Investigating these interactions at the molecular level presents a promising avenue for future research.Therefore, the results of this study serve as a starting point, which should be followed by a thorough scientific study that would reveal the mechanisms of action, synergies between allelochemicals, and the long-term consequences of applying *R. acetosella* into grassland cropping systems. In addition to this, verifying the lab results in the field will be gaining more significance for this data to be used by management in decision-making.

## Data Availability

All data is available within the manuscript. Any other information if required will be made available by the corresponding author on request.

## Abbreviations

ABA: Abscisic acid
JA: Jasmonic acid
SA: Salicylic acid
ROS: Reactive oxygen species
LLE: Liquid–liquid extraction
